# Bioactive Peptides from Skipjack Tuna Cardiac Arterial Bulbs (II): Protective Function on UVB-Irradiated HaCaT Cells through Antioxidant and Anti-Apoptotic Mechanisms

**DOI:** 10.3390/md21020105

**Published:** 2023-02-01

**Authors:** Jing Kong, Xiao-Meng Hu, Wei-Wei Cai, Yu-Mei Wang, Chang-Feng Chi, Bin Wang

**Affiliations:** 1 Zhejiang Provincial Engineering Technology Research Center of Marine Biomedical Products, School of Food and Pharmacy, Zhejiang Ocean University, Zhoushan 316022, China; 2 National and Provincial Joint Laboratory of Exploration, Utilization of Marine Aquatic Genetic Resources, National Engineering Research Center of Marine Facilities Aquaculture, School of Marine Science and Technology, Zhejiang Ocean University, Zhoushan 316022, China

**Keywords:** skipjack tuna (*Katsuwonus pelamis*), antioxidant peptide, skin photoaging, ultraviolet radiation, protective function, anti-apoptosis

## Abstract

The aim of this study was to investigate the protective function and mechanism of TCP3 (PKK), TCP6 (YEGGD) and TCP9 (GPGLM) from skipjack tuna cardiac arterial bulbs on skin photoaging using UVB-irradiated HaCaT cell model. The present results indicated that TCP3 (PKK), TCP6 (YEGGD) and TCP9 (GPGLM) had significant cytoprotective effect on UVB-irradiated HaCaT cells (*p* < 0.001). Hoechst 33342 staining showed that apoptosis of UV-irradiated HaCaT cells could be significantly reduced by the treatment of TCP3 (PKK), TCP6 (YEGGD) and TCP9 (GPGLM); JC-1 staining showed that TCP3 (PKK), TCP6 (YEGGD) and TCP9 (GPGLM) could protect HaCaT cells from apoptosis by restoring mitochondrial membrane potential (MMP); Furthermore, TCP3 (PKK), TCP6 (YEGGD) and TCP9 (GPGLM) could significantly down-regulate the ratio of Bax/Bcl-2 and reduce the expression level of the apoptosis-executing protein Caspase-3 by decreasing the expression of protein Caspase-8 and Caspase-9 (*p* < 0.05). The action mechanism indicated that TCP3 (PKK), TCP6 (YEGGD) and TCP9 (GPGLM) could up-regulate the expression levels of Nrf2, NQO1 and HO-1 (*p* < 0.05), which further increased the activity of downstream proteases (SOD, CAT and GSH-Px), and scavenged reactive oxygen species (ROS) and decreased the intracellular levels of malondialdehyde (MDA). In addition, molecular docking indicated that TCP3 (PKK) and TCP6 (YEGGD) could competitively inhibit the Nrf2 binding site because they can occupy the connection site of Nrf2 by binding to the Kelch domain of Keap1 protein. TCP9 (GPGLM) was inferred to be non-competitive inhibition because it could not bind to the active site of the Kelch domain of Keap1 protein. In summary, the antioxidant peptides TCP3 (PKK), TCP6 (YEGGD) and TCP9 (GPGLM) from cardiac arterial bulbs of skipjack tuna can effectively protect HaCaT cells from UVB-irradiated damage and can be used in the development of healthy and cosmetic products to treat diseases caused by UV radiation.

## 1. Introduction

Skin aging is a complicated bio-process that occurs over time as a result of intrinsic or genetically programmed aging, as well as external aging induced by environment aspect [1,2]. Except for the internal aging process, the sun-exposed body surface including the dorsum of hands, neck, forearms, and face meets with additional destructive effects, mainly because of exposure to ultraviolet light (UV). Photoaging is defined as the influences of prolonged UV radiation and sunshine injury superposed on inherently aged skin [2]. According to the wavelength, ultraviolet radiation can be divided into: UVA, UVB, and UVC with the UV wavelengths of 315–400, 280–315 and 200–280 nm, respectively [3]. Experiments have proved that ozone in the atmosphere can absorb UVC and avoid its damage to human body. Among the UV radiation irradiated to the earth surface, UVB only accounts for 5%, but UVB causes 800–1000 times more damage to the skins than the same dose of UVA. Keratinocytes, as UVB target cells, can receive 95% of the radiation and thus induce photoaging [4]. UVB has stronger mutagenicity and carcinogenicity compared with UVA [5]. UVB radiation directly damages DNA and produces large amounts of reactive oxygen species (ROS) in skin organism [6]. With the overproduction of ROS, the oxidation–reduction dynamic balance in the body is destroyed, and proteins and lipids are oxidized, thus causing oxidative stress with a series of clinical symptoms, including pigmentation disorders, cutis laxa, wrinkles, rough skin surface, inflammation, cell apoptosis, and skin malignant tumor [2,7]. In addition, some skin functions declining with age present a stepped-up trend of decline in photoaging skins [2]. Therefore, the large increase in the aging population and the psychosocial impact of aging skin have created a great demand for effective intervention methods and drugs.

Enzymatic and non-enzymatic cutaneous antioxidants in the skin tissues can protect cells against oxidative stress damage under normal physiological condition [8,9]. However, the body’s antioxidant system cannot remove excess ROS and cells are damaged when the damage degree is beyond the adaptive regulation range of cells [10,11,12]. Then, exogenous antioxidants, such as acorbate, flavonoids, carotenoids, and phenols, can scavenge ROS via stimulating specific signaling pathways in cells to improve the antioxidant ability of cells, which has become a potential method to control UV damage [13,14]. Therefore, UV-irradiated injury and antioxidant constituents have been researched extensively in the areas of food, cosmetic products and medicines.

Recently, bioactive peptides were found in a variety of marine organisms, and many of them have exhibited great potential for adjuvant treatment and prevention of skin photoaging due to their outstanding antioxidant function and anti-apoptosis [10,15,16]. For example, gelatin hydrolysate (AMW 873 Da) from salmon skins could ameliorate UV-induced pathologic alteration of the surface structure and morphology of the skin through inhibiting the depletion of hydroxyproline, decreasing malonaldehyde (MDA) content, improving the levels of antioxidant enzymes and glutathione (GSH), and enhancing the immune regulatory system in photoaging skins [17]. The collagen oligopeptides from chum salmon fish skins could maintain moisture, play antioxidant function, and promote the production of collagen and elastin in UVB-irradiated skin tissue of ICR rats. [18]. ATPGDEG from boiled abalone by-products could protect type I pro collagen and DNA in UVB-induced HaCaT cells via reducing the generation of intercellular ROS, decreasing activities of matrix metalloproteinase-1 (MMP-1), MMP-9, and mitogen-activating the MAPKs and NF-κB signaling [19]. It is very similar that YGDEY from tilapia skin has a therapeutic effectiveness in prevention of UVB-induced cellular damage by mitogen-activating the signaling pathways of MAPK and NF-κB, decreasing ROS level, and increasing intracellular antioxidants [20]. Therefore, marine-derived peptides showed great application value in treating skin photoaging in the future.

Skipjack tuna is the most important raw material for canned aquatic products because of its high catch and low value [21,22,23]. It is worth paying attention that many bioactive peptides were prepared from skipjack tuna and its canning processing by-products [18,24,25,26]. These peptides have shown great application potential in functional food, cosmetics and drugs because of their significant bioactivity, such as ACE inhibitory activity [23,27], radical scavenging activity [12,28,29], and cytoprotective ability on H_2_O_2_-damaged cells [8,18,21]. Elastin hydrolysate of tuna cardiac arterial bulbs was usually used in daily cosmetics. Then, eleven antioxidant peptides, including QGD (TCP1), GEQSN (TCP2), PKK (TCP3), GPQ (TCP4), GEEGD (TCP5), YEGGD (TCP6), GEGER (TCP7), GEGQR (TCP8), GPGLM (TCP9), GLN (TCP10), and GDRGD (TCP11), were purified and identified from its hydrolysate in our previous research, and TCP3 (PKK), TCP6 (YEGGD) and TCP9 (GPGLM) were found to have significantly radical scavenging ability and protective function on H_2_O_2_-damaged DNA and HepG2 cells [30]. Therefore, the objectives of the study were to systematic research the cytoprotective function of TCP3 (PKK), TCP6 (YEGGD) and TCP9 (GPGLM) on UVB-irradiated HaCaT Cells through antioxidant and anti-apoptotic mechanisms.

## 2. Results

### 2.1. Cytoprotection of TCP3 (PKK), TCP6 (YEGGD) and TCP9 (GPGLM) on UVB-Irradiated HaCaT Cells

#### 2.1.1. Establishment of UVB-Irradiated Model of HaCaT Cells

**Figure 1:**
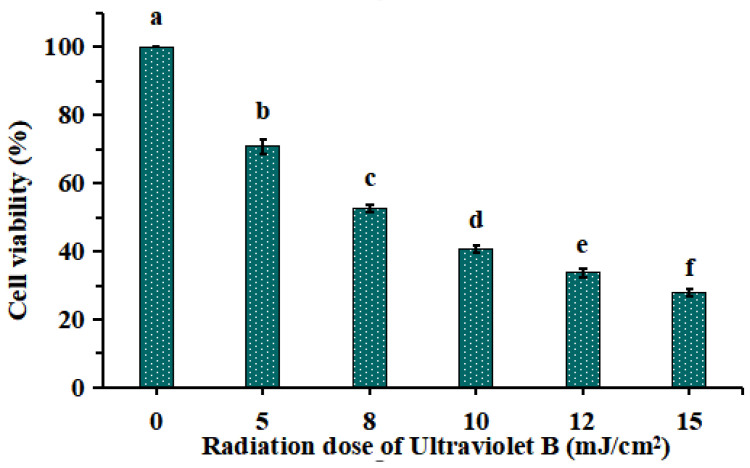
Effects of different ultraviolet B (UVB) radiation doses on the viability of HaCaT cells. a–f Values with same letters indicate no significant difference (*p* > 0.05).

As shown in Figure 1, HaCaT cells were irradiated with different doses of UVB (0, 5, 8, 10, 12 and 15 mJ/cm^2^), and the cell viability was diminished gradually when the doses of UVB were increased from 0 to 15 mJ/cm^2^. The cell viability was 52.52 ± 1.13% at the UVB radiation dose of 8 mJ/cm^2^. As reported by Chen et al. (2016) [19] and Xiao et al. (2019) [20], the optimal radiation dose for establishing UVB injured cell model was decided based on the median lethal radiation intensity. Therefore, 8 mJ/cm^2^ was chosen to the optimal radiation dose for establishing the UVB-irradiated model of HaCaT cells.

#### 2.1.2. Effects of Antioxidant Peptides TCP1-TCP11 on the Viability of UVB-Irradiated Cell Model

**Figure 2:**
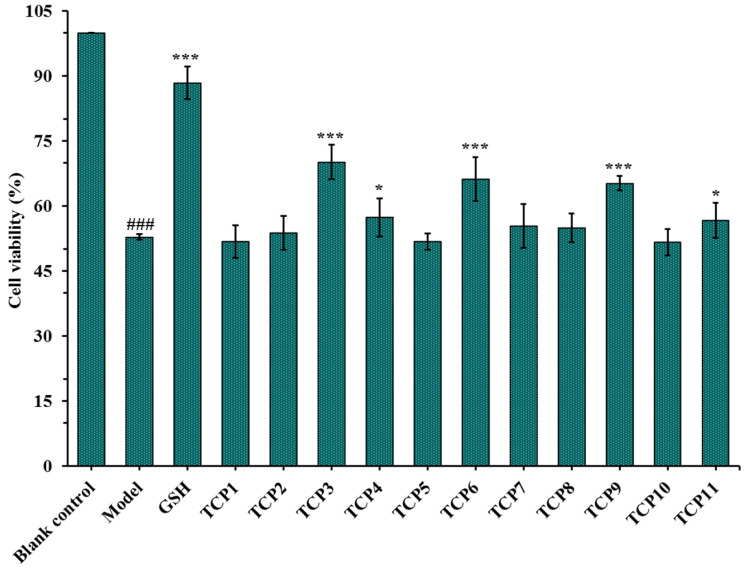
Effects of antioxidant peptides TCP1-TCP11 on the viability of UVB-irradiated cell model. Glutathione (GSH) at 200 µM was served as the positive control. All data are presented as the mean ± SD of triplicate results. ^###^ *p* < 0.001 vs. blank control group; *** *p* < 0.001 and * *p* < 0.05 vs. model group.

After incubated with 200 µM of TCP1-TCP11 and irradiated with 8 mJ/cm^2^ of UVB for 24 h, the cell viability was measured and showed in Figure 2. The results indicated that the cell viability in the model group was 52.82 ± 0.67%, which was significantly lower than that in the blank group (*p* < 0.001). The cell viability of TCP3 (PKK), TCP6 (YEGGD) and TCP9 (GPGLM) groups were 70.15 ± 3.98%, 66.17 ± 5.04% and 65.20 ± 1.66%, respectively, which was significantly higher than those of the model group and other eight peptide groups (*p* < 0.001), suggesting that they could significantly alleviate the oxidative stress damage of HaCaT cells caused by UVB radiation. This finding was in agreement with the previous results that TCP3 (PKK), TCP6 (YEGGD) and TCP9 (GPGLM) exhibited higher antioxidant ability among 11 antioxidant peptides (TCP1-TCP11) [30]. Therefore, the protective function and mechanism of TCP3 (PKK), TCP6 (YEGGD) and TCP9 (GPGLM) on the UVB-irradiated HaCaT cell model will be further discussed.

#### 2.1.3. Effects of TCP3 (PKK), TCP6 (YEGGD) and TCP9 (GPGLM) on the Viability of HaCaT Cells

**Figure 3:**
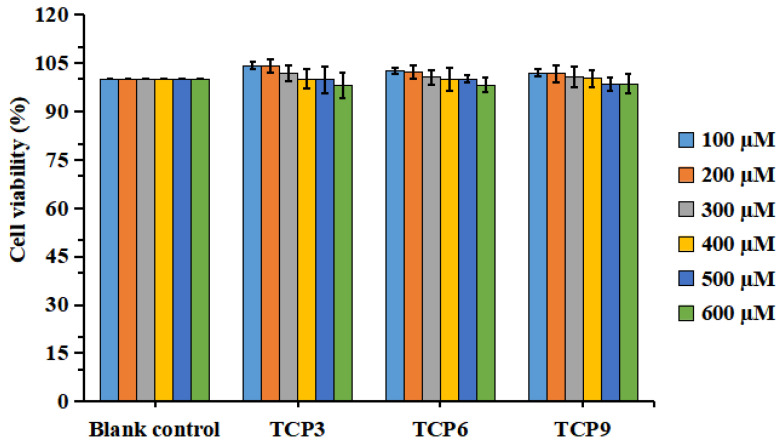
Effects of different concentrations (100–600 μM) of TCP3 (PKK), TCP6 (YEGGD) and TCP9 (GPGLM) on the viability of HaCaT cells. All data are presented as the mean ± SD of triplicate results.

Figure 3 indicated that the viability of HaCaT cells in all determined group was ranged from 98.04 ± 3.94% to 104.32 ± 1.27%. In addition, TCP3 (PKK), TCP6 (YEGGD) and TCP9 (GPGLM) could increase the viability of HaCaT cells at 100-400 µM, but the cell viability was lowered when the peptide concentration exceeded 400 µM. However, no significant difference was found at different concentration (*p* > 0.05). Then, the concentrations of 100, 200, and 400 µM were chosen for further experiment.

#### 2.1.4. Effects of Different Concentrations of TCP3 (PKK), TCP6 (YEGGD) and TCP9 (GPGLM) on the Viability of UVB-Irradiated HaCaT Cell Model

**Figure 4:**
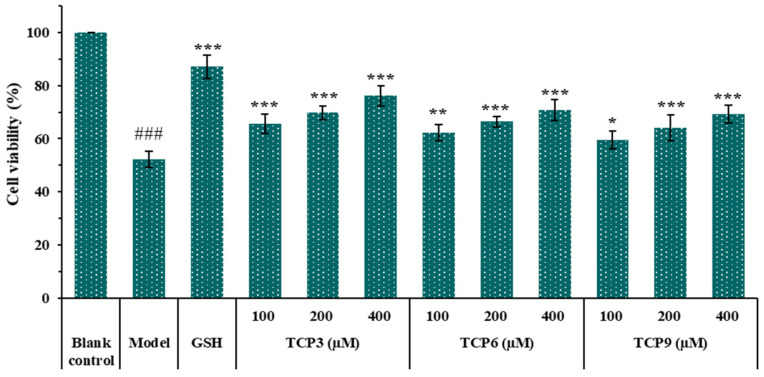
Effects of different concentrations (100, 200, and 400 µM) of TCP3 (PKK), TCP6 (YEGGD) and TCP9 (GPGLM) on the viability of UVB-irradiated cell model. Glutathione (GSH) at 200 µM was served as the positive control. All data are presented as the mean ± SD of triplicate results. ^###^ *p* < 0.001 vs. blank control group; *** *p* < 0.001, ** *p* < 0.01 and * *p* < 0.05 vs. model group.

Figure 4 showed that the cell viability in TCP3 (PKK), TCP6 (YEGGD) and TCP9 (GPGLM) groups increased steadily when the peptide concentration raised from 100 μM to 400 μM. At 400 µM, the cell viability in TCP3 (PKK), TCP6 (YEGGD) and TCP9 (GPGLM) groups was 76.17 ± 3.87%, 70.81 ± 3.86%, and 69.32 ± 3.32%, respectively, which was significantly higher than that of model group (52.20 ± 3.12%)(*p* < 0.001). The data indicated that TCP3 (PKK), TCP6 (YEGGD) and TCP9 (GPGLM) could dose-dependently protect HaCaT cells from UVB damage.

#### 2.1.5. Effects of TCP3 (PKK), TCP6 (YEGGD) and TCP9 (GPGLM) on the ROS Levels of UVB-Irradiated Cell Model

**Figure 5:**
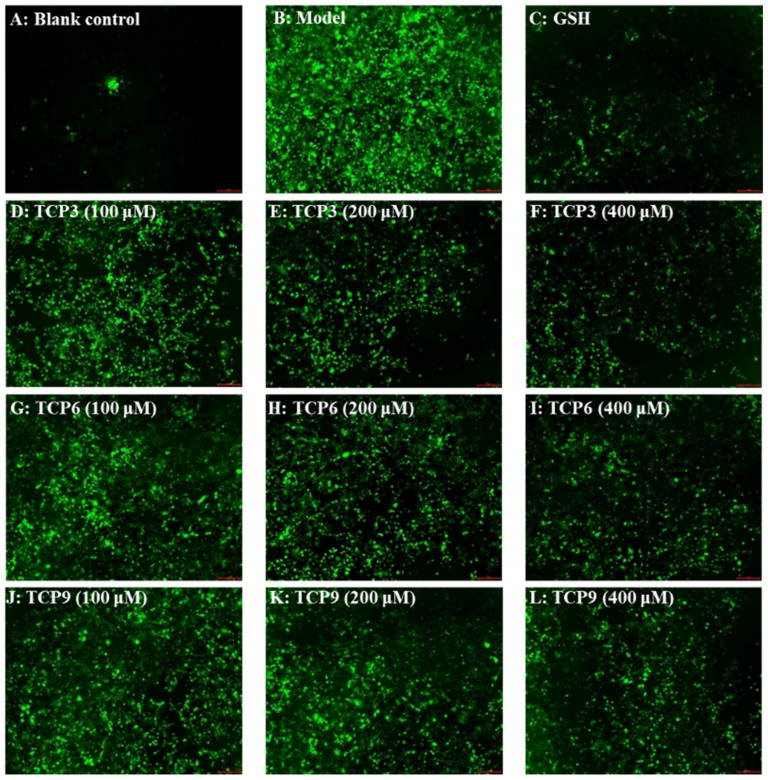
Determination of ROS content in cells by DCFH-DA staining. Glutathione (GSH) at 200 µM served as the positive control. (**A**) Control; (**B**) UVB-irradiated HaCaT cell model; (**C**) GSH; (**D**–**F**) TCP3 with 100, 200, and 400 μM, respectively; (**G**–**I**) TCP6 with 100, 200, and 400 μM, respectively; (**J**–**L**) TCP9 with 100, 200, and 400 μM, respectively.

**Figure 6:**
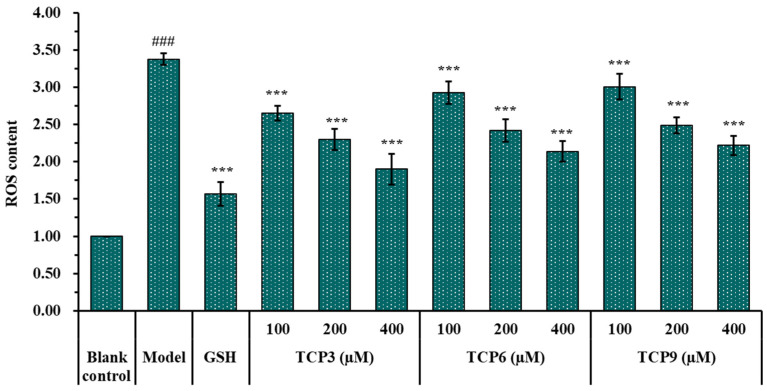
Effects of different concentrations (100, 200, and 400 µM) of TCP3 (PKK), TCP6 (YEGGD) and TCP9 (GPGLM) on ROS levels of UVB-irradiated HaCaT cell model. Glutathione (GSH) at 200 µM was served as the positive control. All data are presented as the mean ± SD of triplicate results. ^###^ *p* < 0.001 vs. blank control group; *** *p* < 0.001 vs. model group.

The influence of TCP3 (PKK), TCP6 (YEGGD) and TCP9 (GPGLM) on the ROS levels in the UVB-damaged model of HaCaT cells was presented in Figure 5 and Figure 6. After DCFH-DA staining, fluorescence intensity and fluorescence area in model group (B) were increased compared with blank group (A), indicating a significant increase in intracellular ROS content. Fluorescence area and intensity of TCP3 (PKK), TCP6 (YEGGD) and TCP9 (GPGLM) groups decreased with the increase of antioxidant peptide concentration compared with the model group, indicating a significant decrease in intracellular ROS content. Figure 6 accurately quantified the influence of TCP3 (PKK), TCP6 (YEGGD) and TCP9 (GPGLM) on ROS levels in the UVB-irradiated HaCaT cells. The ROS levels of TCP3 group at 100, 200 and 400 μM were decreased from 338 ± 8% to 265 ± 10%, 230 ± 14%, and 190 ± 21% of the control group, respectively. The ROS levels of TCP6 group at 100, 200 and 400 μM were decreased from 338 ± 8% to 293 ± 15%, 242 ± 15%, and 214 ± 14% of the control group, respectively. The ROS levels of TCP9 group at 100, 200 and 400 μM were decreased from 338 ± 8% to 301 ± 17%, 249 ± 11%, and 222 ± 13% of the control group, respectively. Therefore, ROS levels were significantly decreased by TCP3 (PKK), TCP6 (YEGGD) and TCP9 (GPGLM) pretreatment at designed concentrations compared with the model group (*p* < 0.001).

#### 2.1.6. Effects of TCP3 (PKK), TCP6 (YEGGD) and TCP9 (GPGLM) on the Intracellular Oxidases and Oxide Levels of UVB-Irradiated HaCaT Cell Model

**Figure 7:**
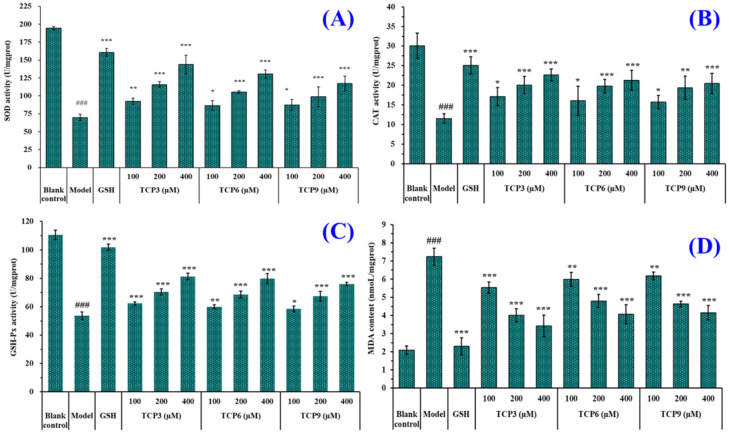
Effects of different concentrations (100, 200, and 400 µM) of TCP3 (PKK), TCP6 (YEGGD) and TCP9 (GPGLM) on the levels of SOD (**A**), CAT (**B**), GSH-Px (**C**), and MDA (**D**) in UVB-irradiated HaCaT cell model. Glutathione (GSH) at 200 µM was served as the positive control. All data are presented as the mean ± SD of triplicate results. ^###^ *p* < 0.001 vs. blank control group; *** *p* < 0.001, ** *p* < 0.01, and * *p* < 0.05 vs. model group.

As shown in Figure 7, the levels of antioxidases (SOD, CAT and GSH-Px) in UVB-irradiated HaCaT cells incubated with TCP3 (PKK), TCP6 (YEGGD) and TCP9 (GPGLM) were gradually increased when the peptide concentrations increased from 100 μM to 400 μM. At 400 μM, the SOD activity in TCP3 (PKK), TCP6 (YEGGD) and TCP9 (GPGLM) groups were 143.82 ± 13.04, 130.43 ± 5.63, and 117.38 ± 10.23 U/mg prot, respectively; the CAT activity in TCP3 (PKK), TCP6 (YEGGD) and TCP9 (GPGLM) groups were 143.82 ± 13.04, 130.43 ± 5.63, and 117.38 ± 10.23 U/mg prot, respectively; the GSH-Px levels in TCP3 (PKK), TCP6 (YEGGD) and TCP9 (GPGLM) groups were 81.28 ± 2.26, 79.77 ± 3.58, and 76.00 ± 1.25 U/mg prot, respectively. The activity of antioxidases in TCP3 (PKK), TCP6 (YEGGD) and TCP9 (GPGLM) groups were significantly higher than those of the model group (*p* < 0.001). Figure 5D showed that the MDA levels of TCP3 (PKK), TCP6 (YEGGD) and TCP9 (GPGLM) groups were decreased dose-dependently with the concentrations of TCP3 (PKK), TCP6 (YEGGD) and TCP9 (GPGLM) increased from 100 μM to 400 μM. At 400 μM, The MDA levels of TCP3 (PKK), TCP6 (YEGGD) and TCP9 (GPGLM) groups decreased to 3.42 ± 0.60, 4.06 ± 0.52, and 4.15 ± 0.39 nmol/mg prot, respectively, which were significantly lower than that of the model group (7.24 ± 0.47 nmol/mg prot) (*p* < 0.001).

### 2.2. Effects of TCP3 (PKK), TCP6 (YEGGD) and TCP9 (GPGLM) on the Apoptosis Rates of UVB-Irradiated HaCaT Cell Model

#### 2.2.1. Effects of TCP3 (PKK), TCP6 (YEGGD) and TCP9 (GPGLM) on the Apoptosis Rates of UVB-Irradiated HaCaT Cell Model

**Figure 8:**
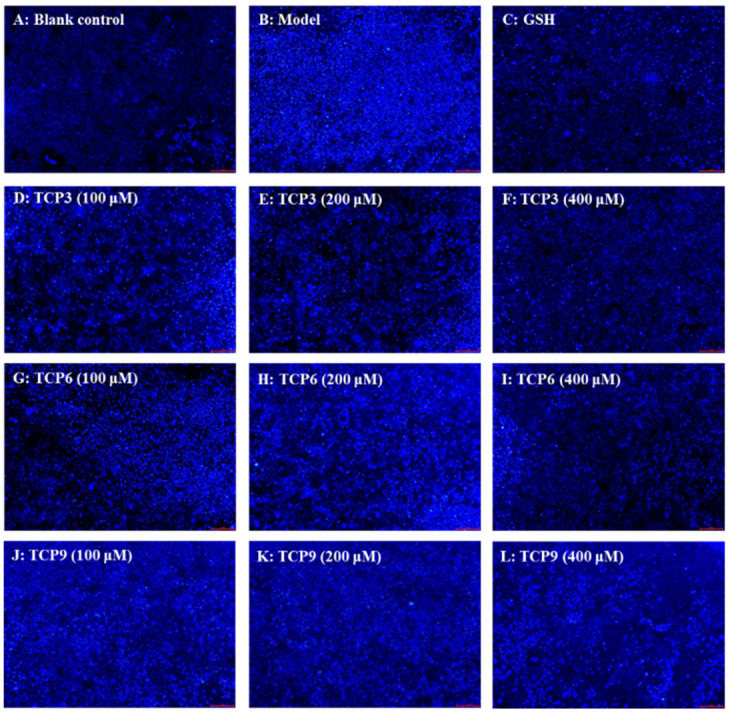
Apoptosis analysis of TCP3 (PKK), TCP6 (YEGGD) and TCP9 (GPGLM) on UVB-irradiated HaCaT cell model by Hoechst 33342. Glutathione (GSH) at 200 µM served as the positive control. (**A**) Control; (**B**) UVB-irradiated HaCaT cell model; (**C**) GSH; (**D**–**F**) TCP3 with 100, 200, and 400 μM, respectively; (**G**–**I**) TCP6 with 100, 200, and 400 μM, respectively; (**J**–**L**) TCP9 with 100, 200, and 400 μM, respectively.

Hoechst 33342 is a solution used to stain the nuclei of living cells and often applied to detect apoptosis. After staining, the nuclei of apoptotic cells were densely or fragmented densely stained. Figure 8A showed that HaCaT cells in the blank group were uniform in size, full in shape, and less burst blue light, but HaCaT cells in the model group (Figure 8B) showed a large amount of blue fluorescence and were in a densely stained state, which indicated a large number of HaCaT cells were damaged by UVB radiation and in an apoptosis state. However, the fluorescence area and intensity of the peptide group gradually decreased with the increase of TCP3, TCP6 and TCP concentrations (Figure 8D–L). In addition, TCP3 showed stronger inhibition than TCP6 and TCP9 on UVB-irradiated HaCaT cell apoptosis. These results illustrated that TCP3 (PKK), TCP6 (YEGGD) and TCP9 (GPGLM) could significantly reduce the apoptosis to protect UVB-irradiated HaCaT cells, which was in agreement with the results in Figure 2 and Figure 4.

#### 2.2.2. Effects of TCP3 (PKK), TCP6 (YEGGD) and TCP9 (GPGLM) on Mitochondrial Membrane Potential (MMP) of UVB-Irradiated HaCaT Cell Model

**Figure 9:**
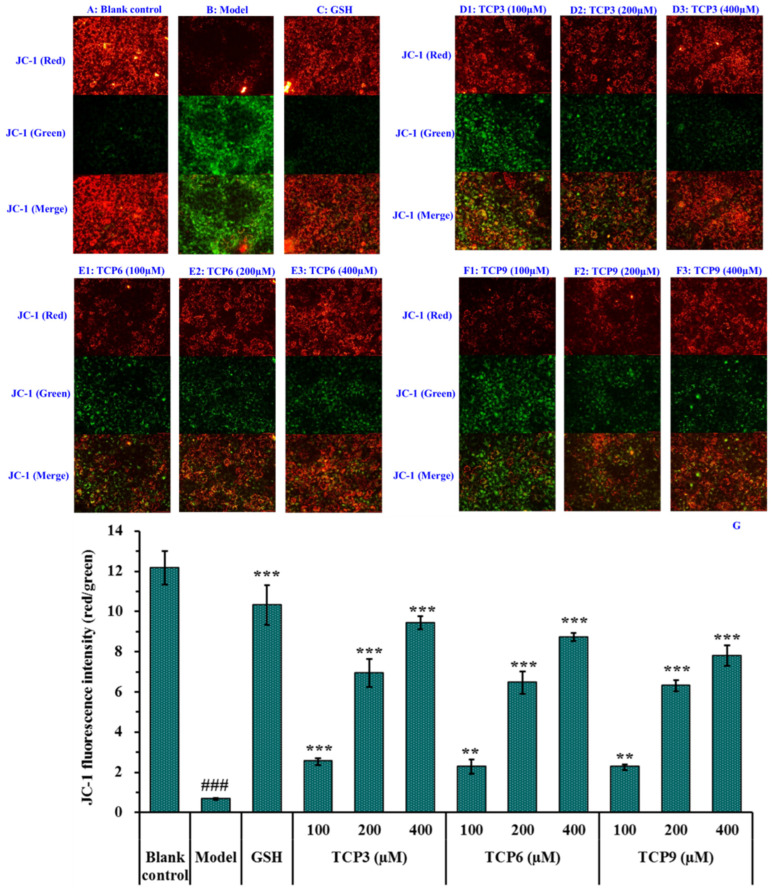
Effects of TCP3 (PKK), TCP6 (YEGGD) and TCP9 (GPGLM) on the mitochondrial membrane potential (MMP) of UVB-irradiated HaCaT cell model. Glutathione (GSH) at 200 µM served as the positive control. (**A**) Control; (**B**) UVB-irradiated HaCaT cell model; (**C**) GSH; (**D1**–**D3**) TCP3 with 100, 200, and 400 μM, respectively; (**E1**–**E3**) TCP6 with 100, 200, and 400 μM, respectively; (**F1**–**F3**) TCP6 with 100, 200, and 400 μM, respectively; (**G**) JC-Fluorescence intensity (red/green). All data are presented as the mean ± SD of triplicate results. ### *p* < 0.001 vs Control group; *** *p* < 0.001, ** *p* < 0.01 vs UVB-irradiated HaCaT cell model.

Mitochondria are the main energy supply units of cells and mitochondrial alterations are one of the most important mechanisms controlling cell apoptosis [31]. MMP is the most reliable indicator of mitochondrial function and can reflect the functional activity of cells, and mitochondrial function can be assessed through monitoring changes in MMP [11,32]. Moreover, the fluorescence intensity of JC-1 can reflect the change degree of MMP (Figure 9). Mitochondria showed red fluorescence at high membrane potential. The reverse is green. The MMP tended to decrease when cells entered the early stage of apoptosis. That is to say, the red fluorescence gradually converted to green fluorescence [33]. 

Compared with the blank group (Figure 9A), the red fluorescence in model group (Figure 9B) decreased, the green fluorescence increased, and the MMP decreased significantly (*p* < 0.001). In addition, the JC-1 fluorescence intensity (red/green) of the model group was 5.51% of the blank group (Figure 9G). These data indicated that UVB irradiation caused the cells in model group to enter the early stage of apoptosis. The decrease of MMP induced by UVB was concentration-dependently restrained when the HaCaT cells were incubated with TCP3 (PKK), TCP6 (YEGGD) and TCP9 (GPGLM) at 100-400 μM (*p* < 0.01) (Figure 9D–F). At 400 μM, the JC-1 fluorescence intensity (red/green) of TCP3 (PKK), TCP6 (YEGGD) and TCP9 (GPGLM) groups was 140.75, 130.30, and 116.42-fold of model group, and TCP3 showed a better increasing function in MMP, which agreed with the results of Figure 8. These finding confirmed that TCP3 (PKK), TCP6 (YEGGD) and TCP9 (GPGLM) could reduce the apoptosis induced by UVB through controlling the decrease of MMP.

### 2.3. Effects of TCP3 (PKK), TCP6 (YEGGD) and TCP9 (GPGLM) on the Expression of Antioxidant and Apoptotic Proteins in UVB-Irradiated HaCaT Cell Model

#### 2.3.1. Expression of Antioxidant-linked Proteins in UVB-Irradiated HaCaT Cell Model

**Figure 10:**
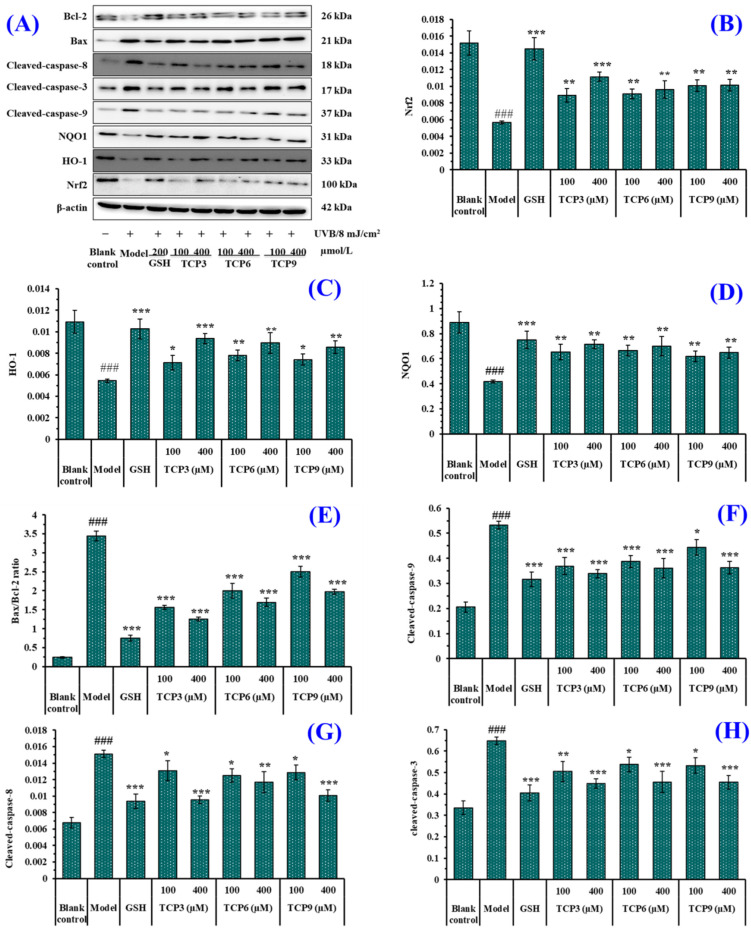
Effect of TCP3 (PKK), TCP6 (YEGGD) and TCP9 (GPGLM) on expression of antioxidant and apoptosis related proteins in UVB-irradiated HaCaT cell model. Glutathione (GSH) at 200 µM was served as the positive control. (**A**) Western-Blot; (**B**) the protein expression of Nrf2; (**C**) the protein expression of HO-1; (**D**) the protein expression of NQO1; (**E**) Bax/Bcl-2 ratio; (**F**) the protein expression of Cleaved-caspase-9; (**G**) the protein expression of Cleaved-caspase-8; (**H**) the protein expression of Cleaved-caspase-3. All data are presented as the mean ± SD of triplicate results. ^###^ *p* < 0.001 vs. control group; * *p* < 0.05, ** *p* < 0.01, and *** *p* < 0.001 vs. UVB-irradiated HaCaT cell model.

The expression of antioxidant-linked proteins including Nrf2, HO-1, and NQO1 were investigated to determine the protective function of TCP3 (PKK), TCP6 (YEGGD) and TCP9 (GPGLM) in the UVB-irradiated HaCaT cell model (Figure 10A). As a transcription factor, Nrf2 can regulate the cellular defense system against oxidative insults by the expression of genes sucked up into oxidative stress response [34,35,36]. As shown in Figure 10B, the protein expression of Nrf2 in the model group was significantly lowered. However, the protein expression level of Nrf2 was apparently recovered after incubating with 100 and 400 μM of TCP3 (PKK), TCP6 (YEGGD) and TCP9 (GPGLM), respectively (*p* < 0.01). At 400 μM, the protein expression level of Nrf2 in TCP3 (PKK), TCP6 (YEGGD) and TCP9 (GPGLM) groups was 1.96-, 1.69-, and 1.79-fold of the model group. It was indicated that TCP3 (PKK), TCP6 (YEGGD) and TCP9 (GPGLM) could activate the Nrf2 pathway, regulating downstream antioxidant enzymes to reduce the UVB damage to HaCaT cells. The finding was verified by the Figure 7A–C that the activity of intracellular antioxidases (SOD, CAT and GSH-Px) in UVB-irradiated HaCaT cells incubated with TCP3 (PKK), TCP6 (YEGGD) and TCP9 (GPGLM) were gradually increased.

HO-1 presents protective effects by metabolizing heme groups to prevent group oxidation or removing ROS by biliverdin and reduced bilirubin. The level of HO-1 is a key indicator to evaluate the antioxidant, anti-inflammatory and anti-apoptosis of drugs [37,38]. As shown in Figure 10C, HO-1 protein expression in the model group was significantly reduced (*p* < 0.001), but the protein expression level of HO-1 was significantly increased with the addition of TCP3 (PKK), TCP6 (YEGGD) and TCP9 (GPGLM) (*p* < 0.001). At 400 μM, the protein expression level of HO-1 in TCP3 (PKK), TCP6 (YEGGD) and TCP9 (GPGLM) groups was 1.72-, 1.65-, and 1.58-fold of the model group. The result proved that TCP3 (PKK), TCP6 (YEGGD) and TCP9 (GPGLM) could protect HaCaT cells against UVB damage by increasing the level of HO-1.

NQO1 is a cytosolic homodimeric flavoprotein that catalyses the two-electron reduction of quinones to reduce the chance of generating reactive oxygen intermediates through the REDOX cycle. NQO1 also maintains α-tocopherol and coenzyme Q10 in a reduced state and protect endogenous antioxidants [39,40]. Figure 10D indicated that the protein expression of NQO1 in the model group was significantly reduced, but the protein expression level of NQO1 in the peptide groups was significantly increased after incubating with TCP3 (PKK), TCP6 (YEGGD) and TCP9 (GPGLM) (*p* < 0.01). At 400 μM, the protein expression level of NQO1 in TCP3 (PKK), TCP6 (YEGGD) and TCP9 (GPGLM) groups was 1.71-, 1.68-, and 1.55-fold of the model group. It was demonstrated that TCP3 (PKK), TCP6 (YEGGD) and TCP9 (GPGLM) could reduce the oxidative damage of HaCaT induced by UV radiation by increasing the expression level of NQO1 protein.

#### 2.3.2. Expression of Apoptosis-linked Proteins in UVB-Irradiated HaCaT Cell Model

The expression of apoptosis-linked proteins including Bax, Bcl-2, caspase 3, caspase 8, and caspase 9 were investigated to determine the protective function of TCP3 (PKK), TCP6 (YEGGD) and TCP9 (GPGLM) in the UVB-irradiated HaCaT cell model (Figure 10A). In the mitochondrial apoptosis pathway, two members of the Bcl protein family (Bax and Bcl-2) play an important role in inhibiting or promoting apoptosis. Mitochondria are the main source of ROS production in keratinocytes exposed to UVB [41]. When ROS content is excessive in mitochondria, the ion concentration on both sides of mitochondrial membrane changes, causing cytochrome C to flow into cytoplasm and form apoptotic bodies with promoter Caspase-9, which then activates apoptotic executive protein Caspase-3, leading to cell apoptosis [42]. In addition, the exogenous pathway is initiated by activation of the death receptor, which is dependent on the protein caspase-8. In the UVB-treated HaCaT cells, the anti-apoptotic Bcl-2 expression was decreased, while the apoptotic Bax expression was increased compared with the control cells (Figure 10A,E). In addition, expression of the cleaved caspase 3, caspase 8, and caspase 9 was highly measured in the UVB-irradiated HaCaT cells (Figure 10A,F–H). However, the expressions of the apoptosis-linked proteins (Bax, Bcl-2, caspase 3, caspase 8, and caspase 9) in TCP3 (PKK), TCP6 (YEGGD) and TCP9 (GPGLM) treated HaCaT cells were reversed, suggesting that TCP3 (PKK), TCP6 (YEGGD) and TCP9 (GPGLM) could promote the expression of Bcl-2/Bax anti-apoptosis protein and down-regulate the expression of cleaved caspase 3, caspase 8, and caspase 9 apoptosis proteins, thus playing their protective role in photoaging HaCaT cells caused by UVB.

### 2.4. Molecular Docking Model of TCP3 (PKK), TCP6 (YEGGD) and TCP9 (GPGLM) with Keap1 Protein

Under normal condition, Nrf2 exists in cytoplasm coupled with Keap1 protein. When ROS are excessive, cysteine residues of Keap1 exposed to ROS are modified, leading to ubiquitination of the Keap1 protein, which interferes with Nrf2 ubiquitination and dissociates Nrf2 from Keap1. In addition, some antioxidant peptides can occupy the binding site of Keap1 and Nrf2, resulting in the dissociation of Nrf2 and Keap1, increasing the amount of free Nrf2 entering the nucleus, and initiating the transcription and translation of downstream antioxidant genes after binding with ARE [43,44].

In Figure 10B, TCP3 (PKK), TCP6 (YEGGD) and TCP9 (GPGLM) can increase the expression level of Nrf2 protein in the nucleus. In order to illustrate the mechanism of TCP3 (PKK), TCP6 (YEGGD) and TCP9 (GPGLM) in the Keap1/Nrf2 pathway, molecular docking method was used to simulate and predict the interactions between TCP3 (PKK), TCP6 (YEGGD) and TCP9 (GPGLM) with Keap1.

**Figure 11:**
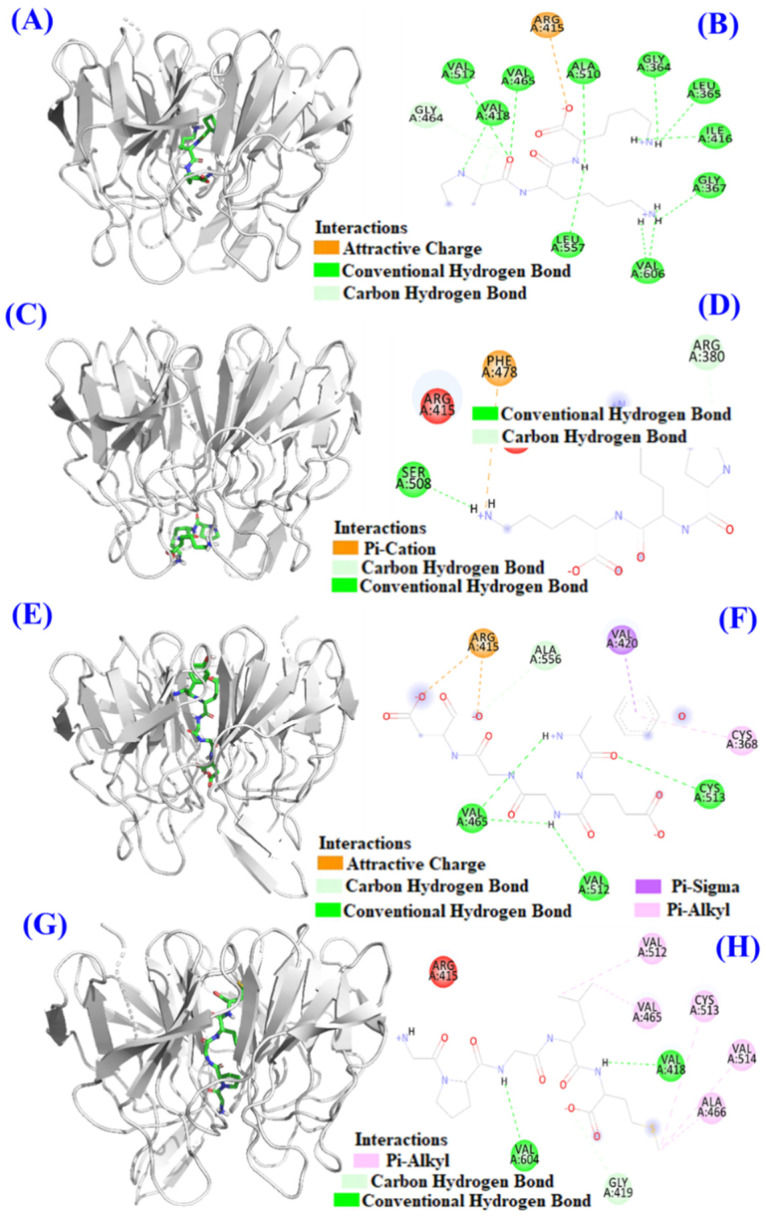
Molecular docking models of TCP3, TCP6, and TCP9 with Keap1 protein. (**A**) 3D details of the middle cavity of the Kelch domain and TCP3 interaction. (**B**) 2D details of the middle cavity of the Kelch domain and TCP3 interaction. (**C**) 3D details of the bottom of the Kelch domain and TCP3 interaction. (**D**) 2D details of the bottom of the Kelch domain and TCP3 interaction. (**E**) 3D details of the middle cavity of the Kelch domain and TCP6 interaction. (**F**) 2D details of the middle cavity of the Kelch domain and TCP6 interaction. (**G**) 3D details of the middle cavity of the Kelch domain and TCP9 interaction. (**H**) 2D details of the middle cavity of the Kelch domain and TCP9 interaction.

Keap1 (MW 70 kDa) is a cysteine-rich protein and consists of more than 625 amino acid residues, including 27 cysteine residues. The Kelch domain of Keap1 is combined with the Neh2 domain of Nrf2 [45,46]. The binding sites of Keap1 in the Kelch domain can be divided into five sub-pockets, namely P1 (Arg415, Ile461, Gly462, Phe478, Arg483 and Ser508), P2 (Ser363, Arg380, Asn382 and Asn414), P3 (Gly509, Ser555, Ala556, Gly571, Ser602 and Gly603), P4 (Tyr525, Gln530 and Tyr572), and P5 (Tyr334 and Phe577) [45,46]. Bioactive peptides bind to other macromolecular substances mainly through hydrogen bond force, van der Waals force and electrostatic interaction force, among which hydrogen bond force is the most important [27,47]. The molecular docking analysis indicated that the affinity of TCP3 with the middle cavity and bottom of Kelch domain was −7.4 kcal/mol and −6.6 kcal/mol, which was similar to those of TCP6 (−8.9 kcal/mol) and TCP9 (−8.5 kcal/mol) interacted with the middle cavity of Kelch domain. These data indicated that TCP3 (PKK), TCP6 (YEGGD) and TCP9 (GPGLM) could bind to Keap1 protein. Figure 11A,B indicated TCP3 (PKK) formed hydrogen bonds with Val418, Val465, Val512, Ala510, Leu557, Gly364, Leu365, Ile416, Val606, Gly367, and Gly464 residues when TCP3 (PKK) interacted at the middle cavity of the Kelch domain and interacted with Arg415 residues of Kelch domain by electrostatic force. In addition, TCP3 (PKK) formed hydrogen bonds with Ser508 and Arg380 residues when TCP3 (PKK) interacted at the middle cavity of the Kelch domain and interacted with Phe478 residues of Kelch domain by electrostatic force (Figure 11C,D). Figure 11E,F showed that TCP6 (YEGGD) formed hydrogen bonds with Cys513, Val465, Val512, and Ala556 residues, interacted with Val420 and Cys368 residues through hydrophobic effect, and acted with Arg415 residue by electrostatic force when it interacted at the middle cavity of the Kelch domain. Figure 11G,H showed that TCP9 (YEGDP) formed three hydrogen bonds with Val418, Val604, and Gly419 residues, and interacted with Ala466, Cys513, Val514, Val512 and Val465 residues through hydrophobic effect when it interacted at the middle cavity of the Kelch domain. In summary, TCP3 (PKK) and TCP6 (YEGGD) occupy the amino acid residues Arg380, Arg415 and Arg415 Ala556 that have an impact on Keap1-Nrf2 interaction, respectively, so it could be concluded that TCP3 and TCP6 can competitively inhibit Nrf2 binding. However, TCP9 (GPGLM) could not bind to the active site of the Kelch domain of Keap1 protein, which was inferred to be non-competitive inhibition.

## 3. Discussion

UV radiation is one of the important environmental factors that causes skin photoaging, which accounts for about 80% of skin aging [48,49,50]. Prolonged skin exposure to UV radiation can induce detrimental intracellular physiological effects and produce superfluous ROS, which can injury intracellular bioactive molecules, such as DNA, enzymes and proteins, and membrane lipids, and further cause oxidative stress and promote cell apoptosis [51,52,53]. Then, inhibiting photoaging induced by UVB can delay skin aging and provides a reasonable basis for studying cosmetic products to treat diseases caused by UV radiation [48]. Therefore, this paper also discusses the protective effects of TCP3 (PKK), TCP6 (YEGGD) and TCP9 (GPGLM) on the cells damaged by UV oxidation from two aspects of antioxidant and apoptosis inhibition. 

Induced by UVB, HaCaT cells produce excessive ROS, which destroy the oxidation-reduction dynamic equilibrium system, and decrease the activities of antioxidant enzymes [54]. With the accumulation of ROS and oxidative metabolites, lipid peroxidation occurs in cells. As one of the end products of lipid peroxidation, MDA can damage the structural and functional integrity of cell membranes [48,53]. The intervention of antioxidant therapy is identified as a potential approach to constrain oxidative stress and improve skin cell function by alleviating ROS damage [50,55]. Therefore, the activity of intracellular antioxidant enzymes and the content of lipid peroxides (MDA) were firstly explored to evaluate the protective effect of TCP3 (PKK), TCP6 (YEGGD) and TCP9 (GPGLM) on the oxidative damage of HaCaT cells after UVB irradiation. The results showed that TCP3 (PKK), TCP6 (YEGGD) and TCP9 (GPGLM) could dose-dependently increase the activity of SOD, CAT and GSH-Px, and reduce the level of MDA in UVB-irradiated HaCaT cells. The results indicated that TCP3 (PKK), TCP6 (YEGGD) and TCP9 (GPGLM) had strong protective effects on UVB-irradiated cells. 

The literature indicated that an increase the level of ROS in cells could negatively affect the Keap1/Nrf2 signaling pathway, thereby bringing down the expression of antioxidant/phase II detoxifying enzymes and leading to oxidative injury and cell apoptosis [31,56]. Protective mechanism indicated that TCP3 (PKK), TCP6 (YEGGD) and TCP9 (GPGLM) could reverse these negative effects by activating Nrf2 pathway to up-regulate the protein expression of Nrf2, HO-1 and NQO1. Oxidative stress can motivate the separation of Nrf2 and Keap1 and accelerate the entry of Nrf2 into the nucleus to bind to the antioxidant response element ARE. Thus, it can promote the expression of antioxidant genes and facilitate the cell REDOX balance [56]. A molecular docking experiment showed that TCP3 and TCP6 occupied the active sites Arg380 and Arg415 of Nrf2 in the Kelch domain of Keap1 protein, while TCP9 did not bind to the active site of Keap1. Therefore, we conclude that TCP3 and TCP6 inhibit Keap1-NrF2 coupling by occupying the active sites of Nrf2 and Keap1 and allow Nrf2 to enter the nucleus and activate pathways to protect cells from oxidative stress. 

Apoptosis is an active reaction of cells after external stimulation, and this process is a form of programmed death regulated by related genes [52,54]. UVB irradiation can cause excessive accumulation of ROS in cells, damage mitochondrial structure and change the permeability of mitochondrial membrane, and mitochondrial alterations are one of the critical paths for manipulating apoptosis [31]. The results of Hoechst 33342 fluorescence staining showed that TCP3 (PKK), TCP6 (YEGGD) and TCP9 (GPGLM) showed inhibitory effects on UVB-induced HaCaT apoptosis. In addition, TCP3 (PKK), TCP6 (YEGGD) and TCP9 (GPGLM) could dose-dependently inhibit the decline of MMP to alleviate cell apoptosis in JC-1 fluorescence double staining assay. In apoptosis, the high Bax/Bcl-2 ratio is a key index in controlling the breakdown of permeability and function of mitochondrial membrane [31,57]. Mechanism of TCP3 (PKK), TCP6 (YEGGD) and TCP9 (GPGLM) inhibiting HaCaT apoptosis suggested that TCP3 (PKK), TCP6 (YEGGD) and TCP9 (GPGLM) could reduce the proportion of Bax/Bcl-2, down-regulate the protein expression levels of caspase 3, caspase 8 and caspase 9, and reverse apoptosis.

Presently, some marine bioactive peptides showed significant protective effect on UV radiation-induced photoaging. Fu et al. reported that collagen peptides from skins and bones of bigeye tuna could reduce the UVB-induced photoaging through regulating MAPK and TGF-β signaling pathways [55]. Peptide fraction from *Pinctada martensii* meat containing oligopeptides FH, AL, MY, AGF, and IYP showed the anti-photoaging activity by increase cell viability, reduced the interstitial MMP-1 and MMP-3 contents, and downregulated the expression of p38, EKR, JNK, MMP-1, and MMP-3 in UVB-induced HaCaT cells [58]. Heptapeptide DAPTMGY from *Isochrysis zhanjiangensis* showed protective effects on HaCaT cells against UVB-induced damage through regulating anti-apoptosis and MAPK/AP-1/MMP pathway [59]. Hydrolysate (TCH) from *Theragra chalcogramma* was rich in GLPYT and could alleviates photoaging via controlling the deposition of collagen fibers and recovery of extracellular component matrix in SD rats [60]. WNLNP from oyster protein hydrolysate had great potential to prevent skin photoaging because it exerted a remarkable antiphotoaging effect on the UVB-irradiated HaCaT cells by regulating MAPK/NF-κB signaling pathway and expression of bax and bcl-2 in UVB-irradiated HaCaT cells [61]. In the study, TCP3 (PKK), TCP6 (YEGGD) and TCP9 (GPGLM) showed a significantly protective function on UVB-damaged HaCaT cells through activating Nrf2 signaling pathways and reducing cell apoptosis. 

## 4. Materials and Methods

### 4.1. Materials and Chemical Reagents

Glutathione (GSP), penicillin–streptomycin solution, phosphate buffered saline (PBS), RPMI modified medium (RPMI-1640), fetal bovine serum, Tris, MTT, and trypsin-EDTA were purchased from Beijing Solabao Technology Co., Ltd. (Beijing, China). Assay kits for determination of the activities of SOD, CAT, and GSH-Px and contents of BCA, ROS and MDA were purchased from Nanjing Jiancheng Bioengineering Institute (Nanjing, China). QGD (TCP1), GEQSN (TCP2), PKK (TCP3), GPQ (TCP4), GEEGD (TCP5), YEGGD (TCP6), GEGER (TCP7), GEGQR (TCP8), GPGLM (TCP9), GLN (TCP10), and GDRGD (TCP11) were synthesized by Shanghai Apeptide Co., Ltd. (Shanghai, China) and their purities were higher than 98%.

### 4.2. HaCaT Cell Culture and Establishment of UV-Irradiated Cell Model

HaCaT cells were bought from the Chinese Academy of Sciences (Shanghai, China) and plated in DMEM supplemented with 12% FBS, streptomycin (100 mg/mL)/penicillin (100 U/mL) at 37 °C in a humidified incubator with 5% CO_2_ [62].

HaCaT cells with the density of 3 × 10^4^ cells/well were seeded into a 96-well plate containing 100 μL of culture media. After 24 h, the culture media was discarded and HaCaT Cells were washed with PBS buffer 3 times. Then, the resulting HaCaT cells were covered with a thin layer PBS and irradiated with different doses of UVB (0, 5, 8, 10, 12, and 15 mJ/cm^2^, respectively) using a UVB (313 nm) light source (Shenzhen Guanya Photoelectric Technology Co., Ltd., Shenzhen, China) with a UVB blocking filter.

Radiation dosage (mJ/cm^2^) = Radiation intensity (mw/cm^2^) × Time (s)

After radiation, HaCaT cells were washed three times with PBS and cultured in new culture media for 24 h. After that, the wells were washed with PBS and MTT was added for an additional 4h. Then, DMSO was added to dissolve the formazan crystals formed by active cells. After that, the absorbance was measured at 570 nm [35,63]. Cell viability was calculated according to the following formula:

Cell viability (%) = (OD_sample_/OD_control_) × 100.

The cell viability was determined and the doses of UVB-induced the HaCaT cell viability of about 50% was chosen to establish the cell model [19,20,27]. 

### 4.3. Effects of Antioxidant Peptides on Cell Viability

After culturing for 24 h, HaCaT cells were treated with 20 µL of peptides (TCP1-TCP11) with the final concentration of 200 µM for 24 h. Then, HaCaT cells were exposed to UVB radiation (8 mJ/cm^2^). After the serum-free medium was incubated for 24 h, cell viability was calculated according to the method in 4.2. The blank group was set without UVB radiation and peptide treatment. The model group was irradiated by UVB without peptide treatment.

In order to research the effects of TCP3 (PKK), TCP6 (YEGGD) and TCP9 (GPGLM) on the UVB-injured cell viability, the final concentration of TCP3 (PKK), TCP6 (YEGGD) and TCP9 (GPGLM) was designed as 100, 200, and 400 µM.

### 4.4. Determination of Intracellular ROS, MDA, and Antioxidases

ROS level in HaCaT cells was monitored according to the previous method [35]. In brief, HaCaT cells were preincubated with peptides at 100, 200, or 400 μM for 12.0 h, and then exposed to UVB (8 mJ/cm^2^). Subsequently, the cells were rinsed by PBS and treated with 10 μM DCFH2-DA in fresh culture medium for 30 min. ROS level indicated by DCF fluorescence were quantified on a BD FACS Calibur flow cytometer.

The activity of SOD, GSH-Px and CAT and content of MDA were measured using assay kits according to the manufacturer’s instructions [64].

### 4.5. Morphological Observation of HaCaT Cells Using Hoechst 33342 Staining Assay

Hoechst 33342 staining assay was performed using previous method [65]. After treating with peptides and UVB radiation, the HaCaT cells were washed, harvested, fixed, and exposed to 8 mg/mL Hoechst 33,342 solution at 37 °C and 5% CO_2_ atmosphere for 30 min. After clearing away the Hoechst 33,342 solution and rinsing three times with serum-free DMEM, the morphology of HaCaT cells was observed using a fluorescence microscope (LSM710; Carl Zeiss Microscopy GmbH, Jena, Germany).

### 4.6. Determination of MMP

MMP was determined using previous method [66]. After treating with peptide and UVB radiation, 100 μL fresh medium and 100 μL JC-1 working medium were added in sequence in the 96-well plate of HaCaT cells. After 40 min, cells were cleaned with PBS and an invertedfluorescence microscopy was employed to capture the fluorescence intensity of HaCaT cells. The intensities of green fluorescence and fluorescence were determined at Ex/Em: 490/530 nm and Ex/Em: 525/590 nm, respectively

### 4.7. Determination of Protein Expression

Western blot was used to measure the protein expression of Bax, Bcl-2, Nrf2, HO-1, NQO1, β-actin, caspase-3, caspase-8, and caspase-9 in HaCaT cells according to our previous method [67]. Total proteins were extracted with a RIPA buffer. After separation of protein with SDS-PAGE, the proteins were transferred into a polyvinylidene difluoride (PVDF) membrane, and the PVDF membrane was blocked with 10% non-immune serum for 2 h. PVDF membranes were incubated with primary antibodies for 12 h at 4 °C and horseradish peroxidaseconjugated secondary antibodies for 2 h at 37 °C. The intensity of the specific immunoreactive bands was determined using enhanced chemiluminescence, quantified by densitometry, and expressed as a ratio to β-actin.

### 4.8. Molecular Docking Experiment of TCP3 (PKK), TCP6 (YEGGD) and TCP9 (GPGLM)

This assay of TCP3 (PKK), TCP6 (YEGGD) and TCP9 (GPGLM) was commissioned to Shanghai NovoPro Biotechnology Co., Ltd. (Shanghai, china). The crystal structure of Keap1 (PDB ID: 2FLU) was acquired from the PDB database. To investigate the possible binding mode of TCP3 (PKK), TCP6 (YEGGD) and TCP9 (GPGLM) to Keap1, the small ligand-binding C-terminal kelch domain of the human Keap1) was selected according to the previous studies [68,69,70]. Molecular docking analysis of TCP3 (PKK), TCP6 (YEGGD) and TCP9 (GPGLM) were carried out in the kelch pockets of Keap1 using AutoDock vina.

### 4.9. Statistical Analysis

All the results are expressed as the mean ± SD (*n* = 3) and analyzed by an ANOVA test using SPSS 19.0. Significant differences between the means of parameters were analyzed by Duncan’s multiple range test (*p* < 0.05).

## 5. Conclusions

In summary, the cytoprotective effects of TCP3 (PKK), TCP6 (YEGGD) and TCP9 (GPGLM) were evaluated against UVB-irradiated HaCaT cells. The cytoprotective mechanisms for TCP3 (PKK), TCP6 (YEGGD) and TCP9 (GPGLM) were the increase in cellular antioxidant capacity through activating Nrf2 signaling pathway and the suppression of cell apoptosis through downregulating Bax-dependent mitochondrial apoptosis. This work laid a theoretical foundation for employing TCP3 (PKK), TCP6 (YEGGD) and TCP9 (GPGLM) to attenuate UVB-irradiated photoaging. In addition, more scientific studies are needed to verify the function of TCP3 (PKK), TCP6 (YEGGD) and TCP9 (GPGLM) in animals to serve as nutraceuticals or functional ingredients in healthy food and cosmetics.

## Data Availability

Data are contained within the article.

## Abbreviations

UV: ultraviolet; ROS, reactive oxygen species; NF-κB, nuclear factor kappa-light chain enhancer of B cells; HaCaT cells, human immortal keratinocyte cell line; Nrf2, nuclear factor erythroid-2-related factor 2; HO-1, heme oxygenase-1; NQO1, nicotinamide quinone oxidore-ductase 1; MTT, methylthiazolyldiphenyl-tetrazolium bromide; GSP, glutathione; SOD, superoxide dismutase; CAT, catalase; GSH-Px, glutathione peroxidase; MDA, malondialdehyde; MAPKs, mitogen-activate protein kinases; DNA, deoxyribonucleic acid; MMP, mitochondrial membrane potential; ACE, angiotensin-I-converting enzyme; DCFH-DA, 2′,7′-Dichlorodihydrofluorescein diacetate; Bax, Bcl-2-associated X-protein; Bcl-2, Proteins of the B-cell lymphoma-2; Keap1, Kelch-like ECH associated protein 1; ARE, antioxidant response elements; TGF-β, Transforming growth factor-β; MMP-1, matrix metalloproteinase-1; MMP-9, matrix metalloproteinase-9; LDH, lactate dehydrogenase; MMP-3, matrix lysing enzyme; MTT, 3-(4, 5-dimethylthiazol-2-yl)-2,5-diphenyltetrazo lium bromide; PVDF: polyvinylidene difluoride.
